# Si-Ni-San alleviates early life stress-induced depression-like behaviors in adolescence via modulating Rac1 activity and associated spine plasticity in the nucleus accumbens

**DOI:** 10.3389/fphar.2023.1274121

**Published:** 2023-11-01

**Authors:** Lihong Ye, Jiayi Wu, Zuyi Liu, Di Deng, Shasha Bai, Lei Yang, Yao Xuan, Zehao Liu, Yafei Shi, Zhongqiu Liu, Rong Zhang, Jinlan Zhao

**Affiliations:** ^1^ Guangdong Provincial Key Laboratory of Translational Cancer Research of Chinese Medicines, Joint International Research Laboratory of Translational Cancer Research of Chinese Medicines, International Institute for Translational Chinese Medicine, School of Pharmaceutical Sciences, Guangzhou University of Chinese Medicine, Guangzhou, China; ^2^ School of Fundamental Medical Science, Guangzhou University of Chinese Medicine, Guangzhou, China

**Keywords:** early life stress, nucleus accumbens, adolescent depression, spine plasticity, Si-Ni-San

## Abstract

**Background:** Early life stress (ELS) is a major risk factor for depression in adolescents. The nucleus accumbens (NAc) is a key center of the reward system, and spine remodeling in the NAc contributes to the development of depression. The Si-Ni-San formula (SNS) is a fundamental prescription for treating depression in traditional Chinese medicine. However, little is known about the effects of SNS on behavioral abnormalities and spine plasticity in the NAc induced by ELS.

**Purpose:** This study aimed to investigate the therapeutic effect and the modulatory mechanism of SNS on abnormal behaviors and spine plasticity in the NAc caused by ELS.

**Methods:** We utilized a model of ELS that involved maternal separation with early weaning to explore the protective effects of SNS on adolescent depression. Depressive-like behaviors were evaluated by the sucrose preference test, the tail suspension test, and the forced swimming test; anxiety-like behaviors were monitored by the open field test and the elevated plus maze. A laser scanning confocal microscope was used to analyze dendritic spine remodeling in the NAc. The activity of Rac1 was detected by pull-down and Western blot tests. Viral-mediated gene transfer of Rac1 was used to investigate its role in ELS-induced depression-like behaviors in adolescence.

**Results:** ELS induced depression-like behaviors but not anxiety-like behaviors in adolescent mice, accompanied by an increase in stubby spine density, a decrease in mushroom spine density, and decreased Rac1 activity in the NAc. Overexpression of constitutively active Rac1 in the NAc reversed depression-related behaviors, leading to a decrease in stubby spine density and an increase in mushroom spine density. Moreover, SNS attenuated depression-like behavior in adolescent mice and counteracted the spine abnormalities in the NAc induced by ELS. Additionally, SNS increased NAc Rac1 activity, and the inhibition of Rac1 activity weakened the antidepressant effect of SNS.

**Conclusion:** These results suggest that SNS may exert its antidepressant effects by modulating Rac1 activity and associated spine plasticity in the NAc.

## Graphical Abstract

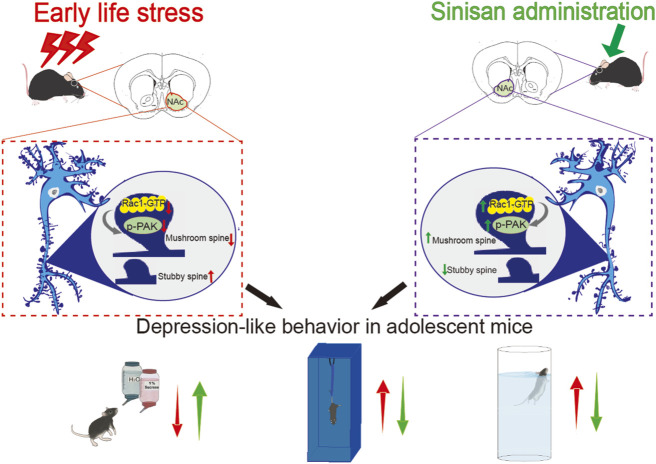

## 1 Introduction

Early Life Stress (ELS) primarily refers to stress experienced from the fetal stage through to puberty. This includes events such as early postnatal mother-infant separation, social isolation in early life, childhood abuse, trauma, neglect, and peer bullying (LeMoult et al., 2020; Zhao et al., 2022). ELS may alter the neural network of the emotional and cognitive systems, making adolescents more prone to the development of psychological and mental disorders (LeMoult et al., 2020). Although most mental disorders typically originate in adolescence, previous studies have largely focused on the impacts of early life stress on adults. Research examining the relationship between early life stress and adolescent depression is only just beginning (LeMoult et al., 2020). Therefore, it is crucial to investigate the mechanisms underlying ELS-induced depression during adolescence.

The primary symptom of adolescent depression is a loss of pleasure, which is tightly linked to dysfunction in the brain’s reward circuit. The Nucleus Accumbens (NAc) serves as the hub for the interaction between dopamine, serotonin, and glutamate, playing a central role in mood and sensory regulation (Zhao et al., 2019). Recent research has demonstrated that early life stress significantly alters the transcription pattern in the NAc, thereby increasing the risk of depression (Pena et al., 2019; Hanson et al., 2021). In addition, it was reported that ELS decreased the response of NAc to rewards in adolescents (Lee et al., 2020). In recent years, synaptic structural remodeling of NAc neurons has been shown to play a critical role in depression. The primary neurons in the NAc (90%–95%) are medium spiny neurons (MSNs), which are projection neurons with numerous dendritic branches and a large number of dendritic spines on the dendrites (Golden et al., 2013; Hanson et al., 2021; Ru et al., 2022). Dendritic spines are tiny protrusions on neuron dendrites and are essential part of excitatory synapses. The structural remodeling of dendritic spines typically includes: 1) changes in spine density; 2) alterations in the shape of spines. Based on their morphology, dendritic spines are primarily divided into three types: stubby spines, thin spines, and mushroom dendritic spines. The size and shape of dendritic spines are closely associated with synaptogenesis and synaptic functional plasticity; as dendritic spines mature from immature (thin and stubby) to mature (mushroom), synaptic strength and stability are enhanced (Golden et al., 2013; Zhao et al., 2019). It has been reported that chronic social defeat stress notably increases the density of stubby spines in NAc MSNs. Alleviating the increase in stubby spine density can improve social avoidance behavior (Gebara et al., 2021), suggesting a close relationship between NAc spine remodeling and depression-like behavior. Despite the importance of NAc spine remodeling in regulating depressive behavior, the impact of early life stress on spine remodeling of NAc in adolescent depression remains unclear.

RAS-related C3 Botulinum Toxin Substrate 1 (Rac1), a key member of the Rho family of small G proteins, plays a significant role in learning and memory, neuropsychiatric disorders, and neuronal synaptic plasticity (Zhao et al., 2019). It has been shown that chronic social stress triggers a reduction in NAc Rac1 expression and that a decrease in Rac1 activity contributes to depression-like behavior and mediates the increase of stubby spines in the NAc (Golden et al., 2013). Our previous studies also discovered that decreased Rac1 activity promoted methamphetamine addiction and increased the density of thin spines in the NAc (Tu et al., 2019; Zhao et al., 2019). However, the role of Rac1 in spine remodeling induced by early life stress remains unknown.

Si-Ni-San (SNS), a formula from the *Shang Han Lun* (Treatise on Febrile Diseases), is a commonly used basic formula in clinical treatment of depression. This formula is composed of four herbs, namely, Chaihu (*Radix Bupleuri*), Baishao (*Radix Paeoniae Alba*), Zhishi (*Fructus Aurantii Immaturus*), and roasted Gancao (*Radix Glycyrrhizae*) (Deng et al., 2022). An advanced study employing Ultrahigh-performance liquid chromatography-high-resolution tandem mass spectrometry (UPLC-HRMS/MS) revealed 713 compounds present in SNS. Remarkably, 13 of these compounds were determined to possess antidepressant properties. These compounds include Trigonelline, Formononetin, Stearic acid, Erucamide, Adenosine, Catechin, Hesperidin, Oleamide, Rutin, Naringin, Vitexin, L-Tyrosine, and Apigenin (Zhang et al., 2023). Additionally, another research identified 37 primary compounds in SNS, with prominent compounds like hesperidin, isoglycyrrhizin, glycyrrhizin, paeoniflorin, and saikosaponin A (Tian et al., 2021), which were also reported to have antidepressant-like effects (Wang et al., 2019; Dai et al., 2022). In addition, a substantial body of research on animals suggests that SNS has antidepressant effect, which can be contributed through the overall regulation of hypothalamus-pituitary adrenocortical system, monoamine neurotransmitters, brain-derived neurotrophic factor and synaptic plasticity (Shen et al., 2020; Wang et al., 2020; Deng et al., 2022). However, most studies have focused on adult depression, and few studies have explored the mechanisms involving the NAc in adolescents (Wang et al., 2020). Targeting synaptic structural plasticity within the NAc could potentially offer new avenues for the treatment of depression, particularly during the critical period of adolescence when the brain is highly malleable. Therefore, it is essential to further investigate the effects of SNS on depression in adolescents and the potential mechanisms regulating NAc spine plasticity in adolescent depression.

The main objective of this study is to examine the therapeutic potential and modulatory mechanisms of SNS in reversing abnormal behaviors and NAc spine plasticity induced by ELS. We hypothesize that SNS may influence depressive behaviors in adolescents exposed to ELS by potentially modulating Rac1 activity and associated dendritic spine dynamics in the NAc. This research holds the potential to elucidate new treatment strategies for adolescent depression, particularly interventions leveraging traditional Chinese medicine.

## 2 Materials and methods

### 2.1 Animals

C57BL/6 pregnant mice, purchased from Southern Medical University (Guangzhou, China) were maintained on a 12-h light/dark cycle with a constant temperature (22°C–24°C). The experiment was conducted in compliance with the Guide for the Care and Use of Laboratory Animals and was approved by the Animal Ethics Committee of Guangzhou University of Chinese Medicine, China (approval No. 2021W0050).

### 2.2 Early life stress paradigm

To assess the impact of ELS on adolescent depression, we utilized an animal model characterized by maternal separation and early weaning at postnatal day 17 (PND17), as established in previous research (Tchenio et al., 2017). Male C57/BL6 mouse pups were arbitrarily divided into two groups: those in the control group and those subjected to early life stress. The early life stress group was subjected to maternal separation from postnatal days 7–15 (PND7-15) and isolated in a new cage equipped with adequate bedding, water, and food for a duration of 6 h per day, with weaning initiated at PND17. Male pups from this group were randomly selected to constitute the ELS group on postnatal day 21 (PND21). On the other hand, the control group pups were left undisturbed until their regular weaning on PND21, after which male pups were randomly assigned to the control group.

### 2.3 Drug preparation and administration

The preparation method and doses calculating are based on our previous (Cao et al., 2019; Deng et al., 2022). SNS, derived from the *Shang Han Lun*, consists of Chai Hu, Shao Yao, Zhi Shi, and roasted Gan Cao in a 1:1:1:1 ratio. Each herb contributes 6 g to the formula. Both the herbal ingredients and fluoxetine procured from the First Affiliated Hospital of Guangzhou University of Chinese Medicine. The preparation of SNS followed traditional modern clinical practices. The herbs were weighed in equal proportions (1:1:1:1 ratio) and were then coarsely ground. The ground herbs were soaked in distilled water at ten times their weight for 60 min. After bringing the solution to a boil, it was simmered for 40 min. The solution was then allowed to cool and was subsequently filtered through an 8-layer cheesecloth to obtain the filtrate. The process was repeated: the herbs were boiled in 8 times the amount of water, simmered, cooled, and filtered. The filtrates from both rounds were combined. This combined solution was concentrated using a rotary evaporator to achieve a concentration of 1.96 g/mL of the original herb. The solution was then aliquoted and stored at 4°C for further use. Considering an average adult body weight of 60 kg/day for dose conversion, the clinically equivalent dose for mice was determined to be 4.9 g/kg. Using a 1:2:4 scaling, the low, medium, and high doses were set at 4.9 g/kg, 9.8 g/kg, and 19.6 g/kg, respectively. The quality and consistency of the SNS preparation were validated using high performance liquid chromatography (HPLC) according to our previous study (Deng et al., 2022). Fluoxetine, SNS and saline (as a vehicle control) were administered via intragastric route from PND22 to PND42. Following the completion of this treatment, behavioral tests were conducted.

### 2.4 High performance liquid chromatography (HPLC)

For liquid chromatographic analysis, 1 mL of SNS solution (1.96 g/mL) was mixed with 23 mL of methanol. The mixture was subjected to ultrasonic radiation for 30 min to ensure uniform dissolution. Subsequently, it was filtered using a 0.45 µm microporous membrane to obtain a clear supernatant. Reference standards were prepared for the following compounds: Hesperidin (B20182, Shanghai Yuanye Bio-Technology Co., Ltd., China), Liquiritin (B20414, Shanghai Yuanye Bio-Technology Co., Ltd., China), Glycyrrhizic acid ammonium salt (IG0740, Beijing Solarbio Science and Technology Co., Ltd., China), Gallic acid (B20851, Shanghai Yuanye Bio-Technology Co., Ltd., China), Paeoniflorin (B21148, Shanghai Yuanye Bio-Technology Co., Ltd., China), Neohesperidin (B21390, Shanghai Yuanye Bio-Technology Co., Ltd., China). Each of these compounds was accurately weighed, dissolved in methanol, and then transferred to individual 1 mL volumetric flasks.

The analysis was performed on an Agilent HPLC-1200 System, utilizing a Diamonsil C18 (2) column (150 × 4.6 mm, 5 μm) maintained at 25°C. The mobile phase comprised of Solution A (acetonitrile) and Solution B (0.01 mol/L aqueous phosphoric acid). The solvent gradients were as follows: 0–10 min: 2%–10% A; 10–20 min: 10%–22% A; 20–28 min: 22%–29% A; 28–40 min: 29%–40% A; 40–50 min: 40%–55% A. Throughout the analysis, the flow rate was consistently maintained at 1 mL/min, and each injection had a volume of 10 μL.

### 2.5 Anxiety-like and depression-like behavior test

Anxiety-like behavior were assessed by open field test (OFT) and the elevated plus maze (EPM), while depression-like behaviors were measured using the sucrose preference test (SPT), the tail suspension test (TST), and the forced swim test (FST) according to our previous study (Zhao et al., 2023). OFT: Each mouse is placed at the center of an open field in a dimly lit room, and their movements are recorded for 10 min using a video camera. This test primarily evaluates the reluctance of the rodent to explore open spaces, which can be indicative of anxiety-like behavior. EPM: This test involves a cross-shaped elevated platform with two open arms and two closed arms. Animals are placed at the center, facing the open-arm direction. Their movement is recorded for 6 min in a dimly lit setting. SPT: To assess anhedonia, a hallmark symptom of depression, mice are initially habituated with two bottles containing a 1% sucrose solution for 2 days. On the third day (test day), they are presented with two bottles-one with 1% sucrose and the other with water-for 24 h. The sucrose preference is calculated as the ratio of the volume of sucrose solution consumed to the total liquid intake (sucrose + water) during the test day, expressed as a percentage. TST: Mice are suspended by the tail using tape, approximately 1 cm from the tail’s tip and 25 cm off the ground. The amount of time the mouse remains immobile over a 6-min period is recorded. FST: Mice are placed in a cylindrical container filled with 25°C water up to a depth of 25 cm. After an initial 6-min period, the duration of immobility is recorded for the subsequent 4 min.

### 2.6 Viral constructs and microinjections

Lentiviruses expressing constitutively active Rac1 driven by the CMV promoter with a double-floxed inverted open reading frame combined with eGFP (DIO-Rac1-CA) and dominant negative Rac1 (DIO-Rac1-DN), or control lentivirus-eGFP (DIO-eGFP) were constructed by Obio Technology Corp., Ltd. Recombinant adeno-associated virus serotype 2/9 (AAV 2/9) expressing mCherry in combination with the Cre enzyme driven by the CMV promoter (CMV-Cre), lentivirus-eGFP (LV-eGFP), and rAAV-hSyn-eGFP-WPRE were constructed by Obio Technology Corp., Ltd. CMV-Cre and DIO-Rac1-CA, DIO-Rac1-DN or DIO-eGFP were bilaterally infused into the NAc over 5 min (coordinates AP, +1.54 mm; ML, ±0.80 mm; and DV,−4.20 mm) according to our previous studies (Tu et al., 2019; Zhao et al., 2019; Ying et al., 2022). Upon Cre-mediated recombination of the DIO, eGFP, Rac1-CA or Rac1-DN are expressed. Rac1-CA simulates the activated state of the wild-type G-protein by mutating glutamine 61 of Rac1 to leucine (Zhang et al., 2006). Rac1-DN inhibits the activity of Rac1 by mutating threonine 17 of Rac1 to asparagine (Tong et al., 2013). In our earlier research, we’ve shown that Rac1-CA can effectively activate Rac1-GTP and its downstream effector, p-PAK, while Rac1-DN inhibits their activity (Zhao et al., 2019).

### 2.7 Dendritic spine analysis of the MSNs

To observe the impact of ELS on the NAc dendritic spines, the virus rAAV-hSyn-eGFP-WPRE was randomly infused into the NAc of both control and ELS mice at PND22. Three mice were used for each group. To investigate the effect of Rac1 on the NAc dendritic spines, the CMV-Cre and either DIO-Rac1-CA, DIO-Rac1-DN, or DIO-eGFP were randomly infused into the NAc of control and ELS mice. Subsequently, based on the type of viral injection, mice were grouped into control + eGFP, ELS + eGFP, control + Rac1-CA, ELS + Rac1-CA, control + Rac1-DN, and ELS + Rac1-DN. After ensuring full viral expression, all groups underwent behavioral tests. After the behavioral test, three mice were randomly selected from each group for dendritic spine analysis. Furthermore, to assess the influence of Si-Ni-San on dendritic spines, three mice were randomly chosen from the control, ELS, positive, and SNS medium dose groups at PND22. The virus rAAV-hSyn-eGFP-WPRE was randomly infused into the NAc of selected mice. Following the behavioral experiments, dendritic spine analysis was conducted. Dendritic spine analysis were performed according to our previous studies (Tu et al., 2019; Zhao et al., 2019; Zhao et al., 2022). The primary antibody was anti-GFP antibody (1:500, Abcam). The second antibody was Alexa Fluor 488-conjugated anti-rabbit antibody (1:200, Invitrogen).

### 2.8 Rac1 activity assay and western blots analysis

The pull-down assay and Western blotting were performed were performed as described before (Zhao et al., 2019). The primary antibodies included the following: anti-Rac1 (1:1000, BD Transduction Laboratories); p-Pak and Pak (1:1000, Cell Signaling); Peroxidase-conjugated goat anti-rabbit or anti-mouse IgG second antibodies (1:5000, Santa Cruz Biotechnology Inc.).

### 2.9 Statistical analysis

Statistical analysis was conducted using SPSS 20.0 software. One-way or two-way ANOVA followed by Bonferroni’s *post hoc* test were employed to evaluate differences among multiple groups, while two groups were performed using Student’s t-tests. Significance was set at *p* < 0.05. Details were described in Supplementary Material.

## 3 Results

### 3.1 Early life stress induced depression-like, not anxiety-like behaviors, in adolescent mice

**FIGURE 1:**
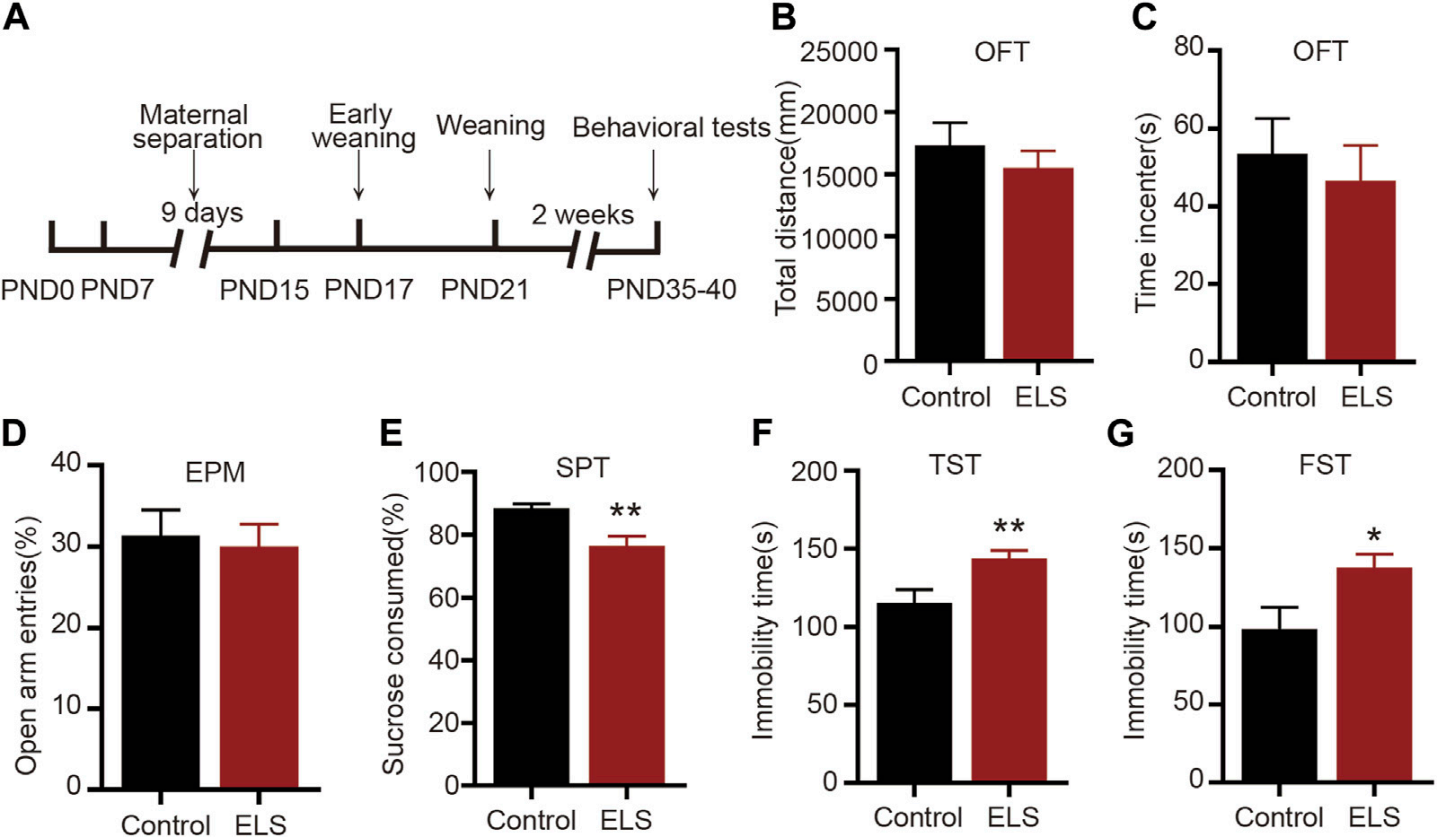
Early life stress induced depression-like, not anxiety-like behaviors, in adolescent mice: **(A)** An experimental paradigm for early life stress. **(B,C)** There was no significant change in the total distance and time spent in the center in the open field test between the two groups (*n* = 14 mice per group). **(D)** In comparison to the control group, there was no significant change in the percentage of open arm entries in the ELS group (*n* = 14 mice per group). **(E)** The percentage of sucrose consumed of the ELS group was significantly decreased (*n* = 14 mice per group). **(F)** Compared with the control group, the immobility time was significantly increased in the ELS group in the tail suspension test (*n* = 14 mice per group). **(G)** Compared with the control group, the immobility time in the forced swimming test was significantly increased in the ELS group (*n* = 14 mice per group). Data were analyzed using Student’s t-test presented as mean ± SEM. **p < 0.01 and *p < 0.05 compared to the control group.

We first evaluated whether early life stress induced depression-like or anxiety-like behavior in adolescent mice. The early-life stress protocol is illustrated in Figure 1A. The OFT and EPM are used to evaluate anxiety-like behavior in adolescent mice. When compared with the control group, the ELS group did not display a significant difference in terms of total distance traversed (Figure 1B, *n* = 14, *p* = 0.427) or time spent in the center during the OFT (Figure 1C, *n* = 14, *p* = 0.591). Similarly, there was no significant difference in the percentage of open arm entries in the ELS group in EPM (Figure 1D, *n* = 14, *p* = 0.761). The FST and TST was used to assess behavioral despair and SPT was used to measure anhedonia-like phenotypes in depression-related behavior. As shown in Figure 1E, the ELS group showed a considerable reduction in the percentage of sucrose intake (Figure 1E, *n* = 14, *p* = 0.001). Additionally, an increase in immobility time was observed in the tail suspension test (Figure 1F, *n* = 14, *p* = 0.007) and the forced swimming test (Figure 1G, *n* = 14, *p* = 0.011) for the ELS group. Taken together, these findings suggest that early life stress primarily induced depression-like, not anxiety-like behaviors, in adolescent mice.

### 3.2 Early life stress induced spine remodeling in the NAc of adolescent mice

**FIGURE 2:**
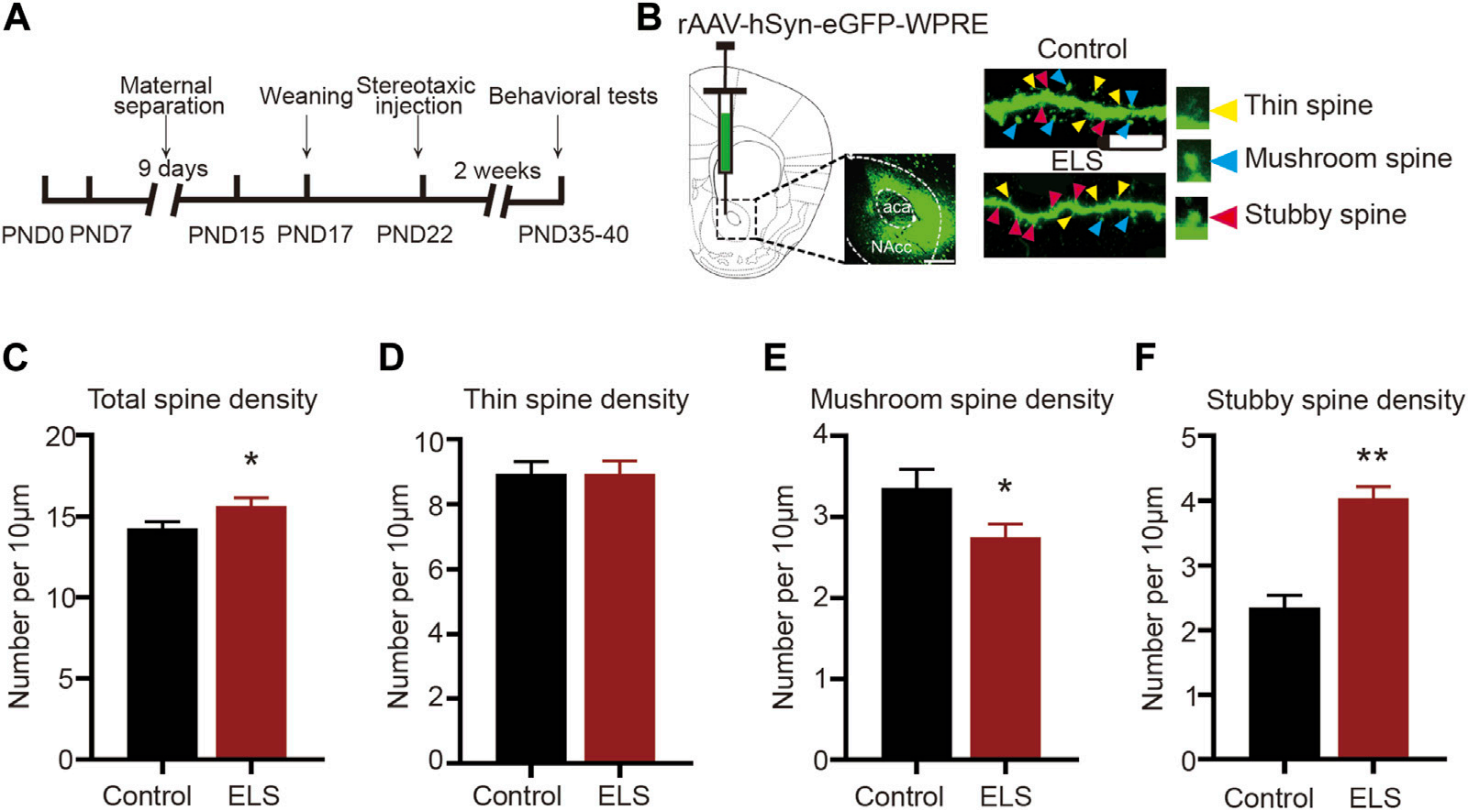
Early life stress induced spine remodeling in the NAc of adolescent mice: **(A)** Anatomical location of the NAc injected with a lentivirus expressing eGFP. Scale bar = 500 µm. **(B)** Representative image of eGFP-labelled spines in the NAc. Scale bar = 10 µm. **(C)** Compared with the control group, the total spine density of the ELS group was significantly increased. **(D)** Compared with the control group, the thin spine density of the ELS group had no significant change. **(E)** Compared with the control group, the mushroom spine density of the ELS group was significantly decreased. **(F)** Compared with the control group, the stubby spine density of the ELS group was significantly increased. 8–12 dendrite sections per animal with 3 animals per group. Data were analyzed using Student’s t-test presented as mean ± SEM. **p < 0.01 and *p < 0.05 compared to the control group.

Chronic stress is known to elicit structural remodeling of dendritic spines. Such alterations can influence the strength and number of synaptic connections between neurons, thereby affecting neural circuits related to mood and emotion regulation (Gebara et al., 2021). To further explore the impact of ELS on the NAc dendritic spines, the virus rAAV-hSyn-eGFP-WPRE was randomly infused into the NAc of both control and ELS mice at PND22. Figures 2A, B schematically represents the area of rAAV-hSyn-eGFP-WPRE injection and the image of eGFP-labelled spines in the NAc. Our findings revealed notable differences in spine morphology between the ELS group and controls. Specifically, compared to the control group, the total spine density was significantly increased in the NAc of ELS group (Figure 2C, 8–12 dendrite sections per animal with 3 animals per group, *p* = 0.033). While thin spine density did not show any significant difference between groups (Figure 2D, *p* = 0.997), we observed a marked decrease in mushroom spine density (Figure 2E, *p* = 0.039) and a substantial increase in stubby spine density (Figure 2F, *p* < 0.001) for the ELS group. These findings suggest that early life stress triggers a restructuring of dendritic spines within the NAc of adolescent mice.

### 3.3 Increased Rac1 activity in the NAc improved early life stress induced-depression-like behaviors

**FIGURE 3:**
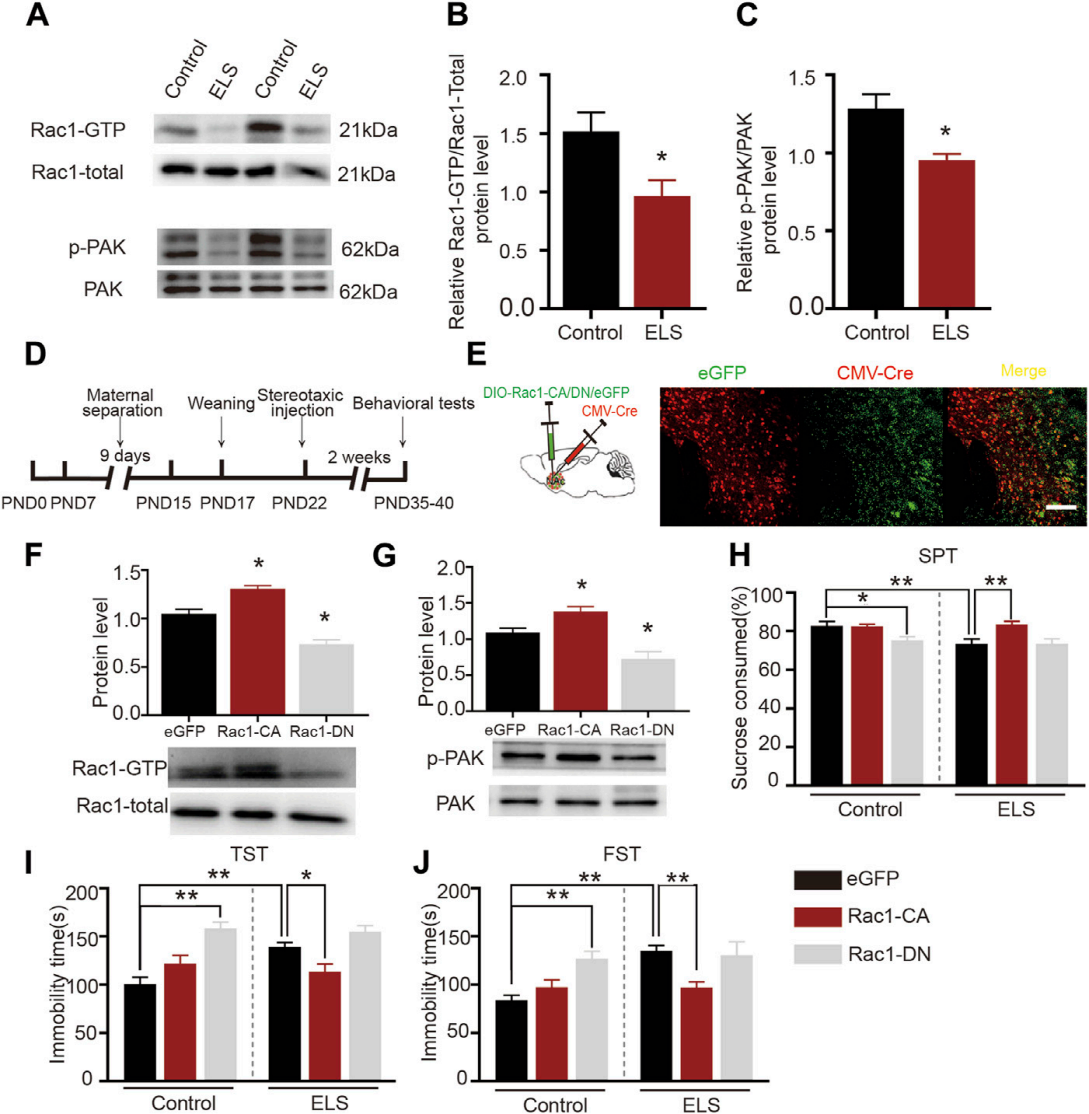
Increased Rac1 activity in the NAc improved early life stress induced-depression-like behaviors: **(A)** Representative band diagram of Rac1-GTP, Rac1-total, p-PAK, PAK protein in the NAc. **(B,C)** Both Rac1-GTPase and its downstream p-PAK activities were decreased in the NAc of the ELS group (*n* = 5 mice per group). **(D)** Diagram of brain area injection. **(E)** Representative images showing colocalization of CMV-Cre virus (red) and eGFP (green) in the NAc. Scale bar = 100 µm. **(F,G)** Western blots for Rac1 and p-Pak activity after the injection Rac1 mutant viruses (*n* = 4 mice per group). **(H)** The effects of Rac1 mutant viruses on the percentage of sucrose consumed (*n* = 8 mice per group). **(I)** The effects of Rac1 mutant viruses on the immobility time in tail suspension test (*n* = 8 mice per group). **(J)** The effects of Rac1 mutant viruses on the immobility time in forced swimming test (*n* = 8 mice per group). Data were analyzed using Student’s t-test **(B,C)**, one-way **(F,G)** or two-way ANOVA **(H–J)** followed by Bonferroni’s *post hoc* test and presented as mean ± SEM. **p < 0.01 and *p < 0.05.

Rac1 plays a significant role in several psychiatric disorders, such as addiction and depressive disorder (Zhao et al., 2019; Ru et al., 2022). We further evaluated the role of Rac1 in ELS induced-depression-like behaviors. AS shown in Figures 3A–C, both Rac1-GTPase (Figure 3B, *n* = 5, *p* = 0.033) and its downstream p-PAK (Figure 3C, *n* = 5, *p* = 0.011) activities were decreased in the NAc of the ELS group compared to the control group. Then, to investigate whether the decrease of Rac1 mediated early life stress induced-depression-like behaviors, CMV-Cre and DIO-Rac1-CA, DIO-Rac1-DN or DIO -eGFP were bilaterally infused into the NAc. Upon Cre-mediated recombination of the DIO, eGFP, Rac1-CA or Rac1-DN are expressed (Figures 3D, E). As shown in Figures 3F, G the constructed Rac1 mutant viruses were able to regulate the activity of Rac1 (Figure 3F, *n* = 4, Rac1-CA vs. eGFP: *p* = 0.043; Rac1-DN vs. eGFP: *p* = 0.01) and its downstream p-Pak activity (Figure 3G, *n* = 4, Rac1-CA vs. eGFP: *p* = 0.044; Rac1-DN vs. eGFP: *p* = 0.01) in the NAc. Moreover, Rac1-CA reversed the low percentage of sucrose consumption observed in the ELS group (Figure 3H, *n* = 8, *p* = 0.001), while Rac1-DN induced a decrease in the percentage of sucrose consumed in the control group (Figure 3H, *p* = 0.03). Similarly, Rac1-CA reversed the increase in immobility time in the ELS group in the tail suspension (Figure 3I, *n* = 8, *p* = 0.038) and forced swimming tests (Figure 3J, *n* = 8, *p* < 0.001), while Rac1-DN induced an increase in immobility time in the control group (Figure 3I, *n* = 8, *p* < 0.001; Figure 3J, *n* = 8, *p* = 0.002). These findings suggest that Rac1 plays a significant role in regulating behaviors associated with depression that result from early life stress.

### 3.4 Increased Rac1 activity in the NAc improved early life stress induced-spine abnormalities in the NAc

**FIGURE 4:**
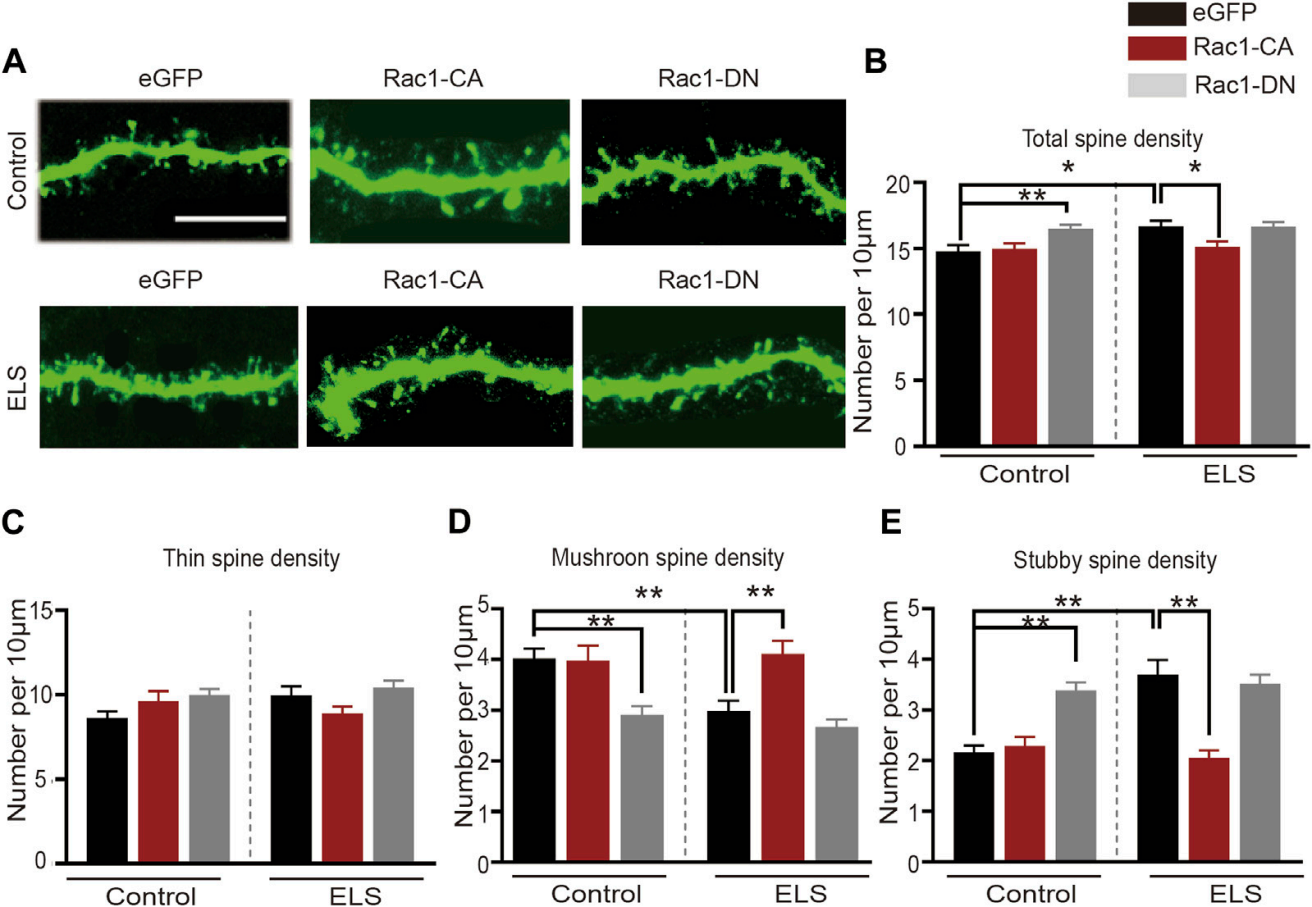
Increased Rac1 activity in the NAc improved early life stress induced-spine abnormalities in the NAc: **(A)** representative confocal images of GFP-labeled dendritic spines in the NAc. Scale bar = 10 µm. **(B)** The effects of Rac1 mutant virus on the total spine density in the NAc. **(C)** The effects of Rac1 mutant virus on thin spine density in NAc. **(D)** The effects of Rac1 mutant virus on the mushroom spine density in the NAc. **(E)** The effects of Rac1 mutant virus on the stubby spine density in the NAc. 8–12 dendrite sections per animal with 3 animals per group. Data were analyzed using two-way ANOVA followed by Bonferroni’s *post hoc* test and presented as mean ± SEM. **p < 0.01 and *p < 0.05.

Rac1 plays a significant role in spine plasticity (Zhao et al., 2019). Therefore, we further examined its role in the spine abnormalities in the NAc induced by ELS. Figure 4A depicted a representative image of the dendrites of neurons in the NAc. We discovered that the overexpression of Rac1-CA significantly decreased the total spine density in the NAc that was induced by early life stress (Figure 4B, 8–12 dendrite sections per animal with 3 animals per group, *p* = 0.0.021), while the overexpression of Rac1-DN on its own resulted in a significant increase in total spine density, mirroring the effect of early life stress on spine remodeling in the NAc (Figure 4B, *p* = 0.009). As shown in Figure 4C, Rac1 did not affect the density of thin spines. Moreover, the reduction in mushroom spine density in the early life stress group was reversed by the Rac1-CA virus (Figure 4D, *p* = 0.001), and Rac1-DN was able to reduce mushroom spine density in the control group (Figure 4D, *p* < 0.001). Additionally, overexpression of Rac1-CA significantly decreased stubby spine density in the NAc induced by early life stress (Figure 4E, *p* < 0.001), whereas the overexpression of Rac1-DN led to a significant increase in stubby spine density in the NAc compared to the control group (Figure 4E, *p* < 0.001). All these findings suggest that a decrease in Rac1 activity in the NAc plays an important role in spine remodeling in the NAc induced by ELS.

### 3.5 Administration of SNS improved depressive-like behavior and spine abnormalities in the NAc induced by early life stress

**FIGURE 5:**
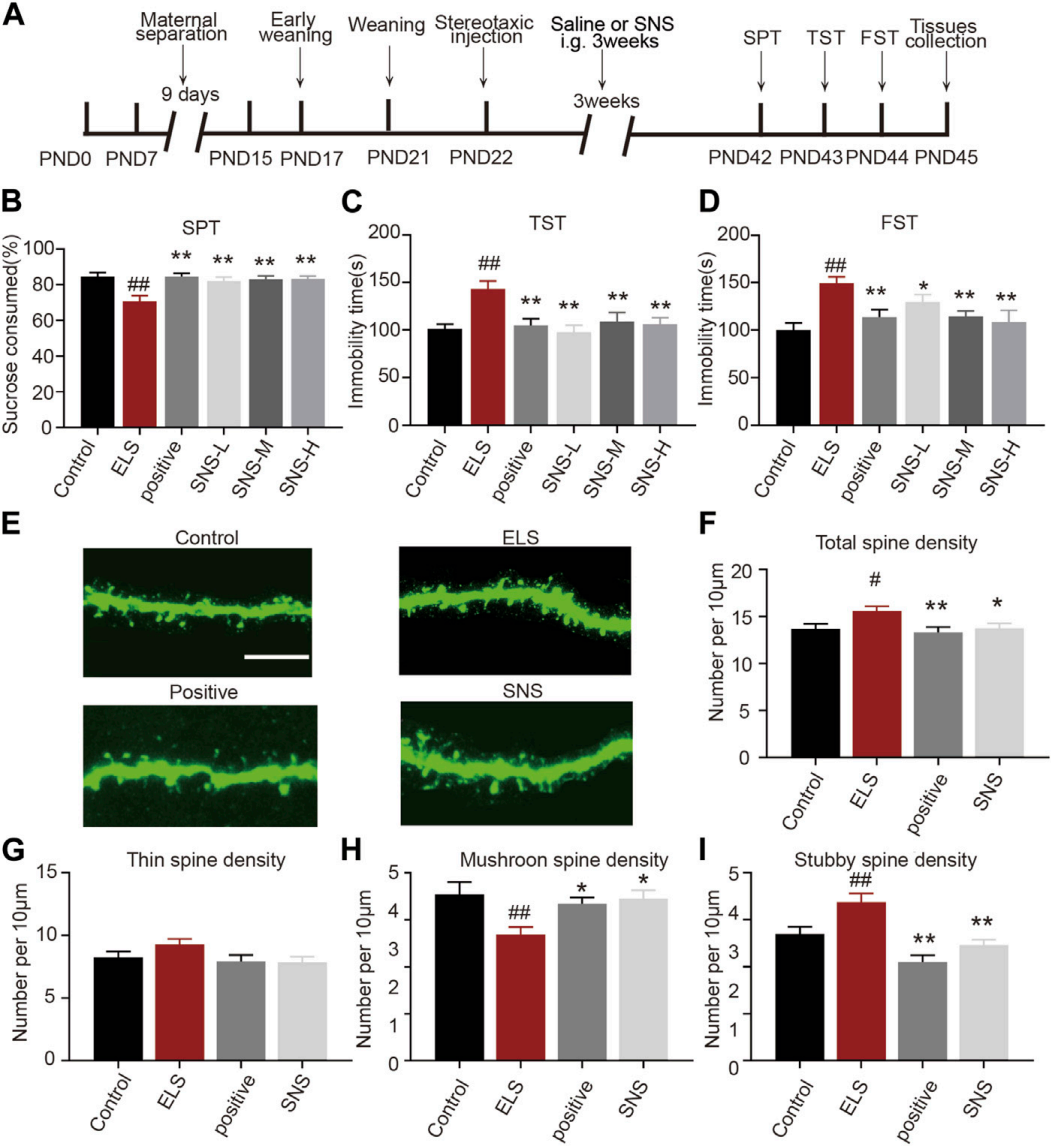
Administration of SNS improved depressive-like behavior and spine abnormalities in the NAc induced by early life stress: **(A)** Schematic diagram of the experimental flow. **(B)** The effects of SNS on the percentage of sucrose consumed (*n* = 14 mice per group). **(C)** The effects of SNS on the immobility time in tail suspension test (*n* = 14 mice per group). **(D)** The effects of SNS on the immobility time in forced swimming test (*n* = 14 mice per group). **(E)** Representative confocal images of GFP-labeled dendritic spines in the NAc. Scale bar = 10 µm. **(F)** The effects of SNS on the total spine density in the NAc. **(G)** The effects of SNS on the thin spines density in the NAc. **(H)** The effects of SNS on the mushroom spine density in the NAc. **(I)** The effects of SNS on the stubby spine density in the NAc. 8–12 dendrite sections per animal with 3 animals per group. Data were analyzed using one-way ANOVA followed by Bonferroni’s *post hoc* test and presented as mean ± SEM. ##*p* < 0.01 and #*p* < 0.05 compared to the control group, **p < 0.01 and *p < 0.05 compared to the ELS group.

A substantial body of research on animals suggests that SNS and its modified prescriptions have an antidepressant effect, which may be attributed to the overall regulation of the hypothalamus-pituitary-adrenal system, monoamine neurotransmitters, brain-derived neurotrophic factor, and synaptic plasticity (Shen et al., 2020; Deng et al., 2022). However, the antidepressant mechanism of SNS is rarely designed for the spine remodeling in the NAc. Therefore, we further investigated whether SNS could alleviate ELS-induced depression-like behaviors and spine abnormalities in the NAc. As shown in Figure 5A, SNS was administered intragastrically at the dose of at 4.9 g/kg (low dose group), 9.8 g/kg (medium dose group), and 19.6 g/kg (high dose group). We found that SNS exerted a significant antidepressant effect. All three doses of SNS significantly increased the percentage of consumed sucrose (Figure 5B, *n* = 14, ELS vs. Control: *p* < 0.001; Positive vs. ELS: *p* < 0.001; SNS-L vs. ELS: *p* = 0.002; SNS-M vs. ELS: *p* = 0.001, SNS-H vs. ELS: *p* = 0.001). Furthermore, all three doses of SNS decreased immobility time in both the tail suspension (Figure 5C, *n* = 14, ELS vs. Control: *p* = 0.001; Positive vs. ELS: *p* = 0.002; SNS-L vs. ELS: *p* < 0.001; SNS-M vs. ELS: *p* = 0.008; SNS-H vs. ELS: *p* = 0.001) and forced swimming experiments (Figure 5D, *n* = 14, ELS vs. Control: *p* < 0.001; Positive vs. ELS: *p* = 0.007; SNS-L vs. ELS: *p* = 0.025; SNS-M vs. ELS: *p* = 0.009; SNS-H vs. ELS: *p* = 0.002). These results suggest that SNS could improve adolescent depression-like behavior induced by ELS. We also evaluated whether SNS could ameliorate spine deficits in the NAc induced by early life stress. Figure 5E presents representative confocal images of dendritic spines in the NAc. We found that SNS reversed the increase in total spine density in the NAc induced by early life stress (Figure 5F, *n* = 4, ELS vs. Control: *p* = 0.028; Positive vs. ELS: *p* = 0.007; SNS vs. ELS: *p* = 0.035). Moreover, SNS had no effect on the density of thin spines in the NAc (Figure 5G, *n* = 4, ELS vs. Control: *p* = 0.356; Positive vs. ELS: *p* = 0.131; SNS vs. ELS:*p* = 0.092). Additionally, SNS administration significantly increased mushroom spine density (Figure 5H, *n* = 4, ELS vs. Control: *p* = 0.006; Positive vs. ELS: *p* = 0.048; SNS vs. ELS: *p* = 0.014) and decreased stubby spine density (Figure 5I, *n* = 4, ELS vs. Control: *p* = 0.005; Positive vs. ELS: *p* < 0.001; SNS vs. ELS: *p* < 0.001) in the NAc induced by ELS. These results indicate that SNS may alleviate depression-like behaviors in adolescence by modulating spine plasticity in the NAc.

### 3.6 The effects of Rac1 signalling on the antidepressant action of SNS in adolescence

**FIGURE 6:**
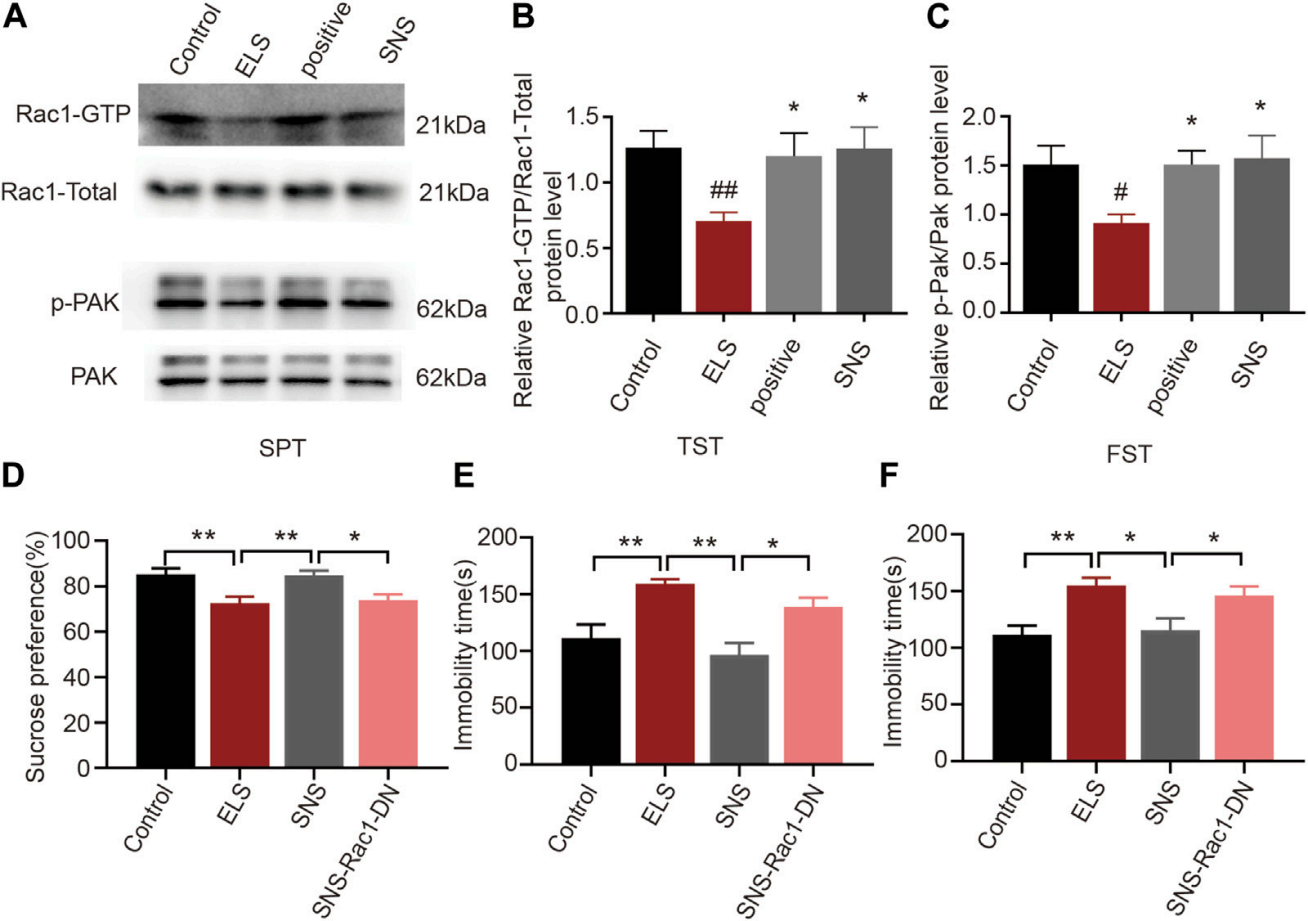
The effects of Rac1 signalling on the antidepressant action of SNS in adolescence: **(A)** Representative band diagram of Rac1-GTP, Rac1-total, p-PAK, PAK protein in the NAc. **(B,C)** Effects of SNS on the Rac1 and p-PAK activity in the NAc (*n* = 4 mice per group). **(D)** Rac1-DN weakened the antidepressant effect of SNS on the percent of sucrose consumed in sucrose preference test (*n* = 8 mice per group). **(E)** Rac1-DN weakened the antidepressant effect of SNS on the immobility time in tail suspension test after SNS administration (*n* = 8 mice per group). **(F)** Rac1-DN weakened the antidepressant effect of SNS on the immobility time in forced swimming test (*n* = 8 mice per group). Data were analyzed using one-way ANOVA followed by Bonferroni’s *post hoc* test and presented as mean ± SEM. **p < 0.01 and *p < 0.05.

Given the established association between Rac1 activity in the NAc and depression-like behaviors, as well as spine remodeling due to early life stress, we further investigated Rac1’s involvement in the antidepressant effects of SNS. We initially measured the impact of SNS on Rac1 activity in the NAc. As demonstrated in Figure 6A, SNS significantly increased Rac1 (Figure 6B, *n* = 4, ELS vs. Control: *p* = 0.004; Positive vs. ELS: *p* = 0.023; SNS vs. ELS: *p* = 0.016) and its downstream p-PAK activity (Figure 6C, *n* = 4, ELS vs. Control: *p* = 0.022; Positive vs. ELS: *p* = 0.035; SNS vs. ELS: *p* = 0.015). Furthermore, we found that overexpression of Rac1-DN significantly weakened the antidepressant effect of SNS, as shown by the decrease in the percentage of consumed sucrose (Figure 6D, *n* = 8, ELS vs. Control: *p* = 0.005; SNS vs. ELS:*p* = 0.007; SNS vs. SNS-Rac1-DN: *p* = 0.016), and the increase in immobility time in both the tail suspension (Figure 6E, *n* = 8, ELS vs. Control: *p* = 0.004; SNS vs. ELS: *p* < 0.001; SNS vs. SNS-Rac1-DN: *p* = 0.018) and forced swimming tests (Figure 6F, *n* = 8, ELS vs. Control: *p* = 0.005; SNS vs. ELS: *p* = 0.011; SNS vs. SNS-Rac1-DN: *p* = 0.038). These results suggest that the antidepressant action of SNS is mediated, at least in part, by modulating Rac1 activity within the NAc.

## 4 Discussion

In this study, our findings revealed that ELS significantly induced depression-like behaviors in adolescent mice. Additionally, ELS increased the stubby spine density while decreasing mushroom spine density in the NAc. Furthermore, ELS reduced Rac1 activity in the NAc, and the overexpression of a constitutively active Rac1 in the NAc reversed depression-related behaviors and led to a decrease in stubby spine density and an increase in mushroom spine density, suggesting that Rac1 plays a crucial role in the behavioral and spine abnormalities induced by ELS during adolescence. Moreover, SNS mitigated depression-like behavior in adolescent mice and counteracted the spine abnormalities in the NAc induced by ELS. Additionally, SNS increased NAc Rac1 activity, and the overexpression of a dominant-negative Rac1 weakened the antidepressant effect of SNS. Our results suggest that SNS may exert its antidepressant effects by modulating Rac1 activity and associated spine plasticity in the NAc.

Maternal separation, social isolation, and other forms of ELS significantly increase risk for depression. Previous studies have largely focused on the impacts of ELS on adults (Hanson et al., 2021; Waters and Gould, 2022). For instance, ELS from PND10–20 changed transcriptomic patterning in the brain’s reward circuitry and increased the susceptibility to depression-like behavior in adult mice (Pena et al., 2019). In our study, we used an ELS animal model of maternal separation with early weaning to evaluate the effects of ELS on behavioral abnormalities in adolescence. The ELS paradigm was based on both rodent maternal separation studies and work demonstrating early weaning reduced compensatory maternal care after maternal separation (Tchenio et al., 2017; Waters and Gould, 2022). In this study, we found that ELS induced depression-like behavior in adolescence which is in accordance with previous study (Tchenio et al., 2017; Alteba et al., 2021; Chen et al., 2021). A study has shown that maternal separation combined with early weaning induced anxiety-like behavior in rats (Zeng et al., 2020). The discrepancy between their findings and ours might arise from species differences. This speculation is substantiated by research indicating that the effects of early life stress on depression and anxiety-like behaviors can be both species and gender-specific (Zeng et al., 2021). Another study reported that maternal separation with early weaning induced anxiety, hyperactivity, and behavioral despair in CD1 mice (Gracia-Rubio et al., 2016). Importantly, it is noteworthy that many experiments involving C57BL/6J male mice have yielded divergent outcomes concerning depression and anxiety-like behaviors (Tan et al., 2017). The manifestation of depression-like behaviors without concurrent anxiety-like behaviors in our study might be attributed to variances in the maternal separation protocol. Indeed, research has shown that the consequences of the maternal separation protocol depend on the duration, developmental stage, and number of days of the separation experience (Nishi, 2020).

NAc is a key brain region involved in reward processing and motivation. It plays a crucial role in the development of depressive symptoms (Golden et al., 2013; Zhao et al., 2022). A recent study suggested that ELS induced enduring transcriptional changes in the NAc that may underlie vulnerability to stress in adulthood (Pena et al., 2019). Previous studies investigating the relationship between spine plasticity and stress have been conducted in the context of social stress, but not in the context of early life stress (Warren et al., 2014; Lee et al., 2020). It is not yet known how ELS alters NAc spine plasticity in adolescence. In this study, we found that ELS increased total density of dendritic spines, especially those of stubby spines of the NAc in male mice. Additionally, ELS decreased mushroom spine density in the NAc. Our study was consistent with previous study demonstrating that emotional stress and physical stress increased spine density in the NAc of adolescent exposed mice (Warren et al., 2014). Similarly, it was reported that high-trait-anxiety rats showed more thin spines and fewer mushroom spines in the NAc (Gebara et al., 2021). Our previous study also demonstrated that adolescent social isolation induced anxiety-like behavior and increased thin spine density in the NAc (Zhao et al., 2022). To our knowledge, this study is the first to evaluate the effect of ELS on spine plasticity in the NAc during adolescence. Spine remodeling provides the structural basis for functional changes in neural circuits. Stubby spines are relatively short and are thought to represent newly formed or less stable synapses, and they are more dynamic and can undergo changes in density and shape. Mushroom spines are considered mature and stable, representing well-established and strong synapses, and are associated with long-term synaptic potentiation (Runge et al., 2020; Zhao et al., 2022). Previous study also reported that chronic social defeat stress notably increases the density of stubby spines in NAc MSNs (Golden et al., 2013). In addition, it was reported that increased spine density was associated with hyperexcitability of neurons (Ru et al., 2022). Thus, we speculate that increased stubby spine density and decreased mushroom spine density in the NAc may reflect heightened sensitivity to stress in adolescence. A recent study also demonstrated that adolescents with major depressive disorder showed increased NAc volume, which was significantly correlated with depressive symptoms in adolescence (Lee et al., 2020). In addition, it was reported that dendritic spine density was associated with brain volumetric changes (Keifer et al., 2015). Taken together, these findings indicate that NAc spine density may be one possible structural alteration that plays an important role in adolescent depression.

Rac1 is a small GTPase protein that plays a crucial role in regulating actin cytoskeleton dynamics and cellular processes such as spine, and synapse development. Over time, evidence has suggested that Rac1 plays a significant role in several psychiatric disorders, such as addiction and depressive disorder (Zhao et al., 2019; Ru et al., 2022). However, the protective role of Rac1 has not yet been examined in an animal model of adolescent depression. Our results showed that maternal separation combined with early weaning could decrease Rac1 activity, whereas increased Rac1 activity could improve depressive-like behavior and prevent spine abnormalities in the NAc caused by early life stress. This is consistent with our previous studies demonstrating that Rac1 attenuated behavioral and spine abnormalities in the NAc induced by methamphetamine (Zhao et al., 2019). Additionally, it is worth noting that previous evidence from the social defeat mice model showed that malvidin-3′-O-glucoside exerts an antidepressant effect by enhancing Rac1 expression in the NAc (Wang et al., 2018). Taken together, our evidence further supports the important role of rac1 in spine remodeling and the treatment of depression, especially in adolescent depressive patients who have experienced early life stress.

SNS was first documented in Zhang Zhongjing’s Treatise on Febrile Diseases. It has been traditionally considered a classic formula for soothing the liver and alleviating depression, and showed many advantages in the treatment of depression (Deng et al., 2022). Our previous studies have demonstrated that SNS delivers anti-depressive effects by regulating hippocampal synaptic plasticity, mitochondrial function, and brain-derived neurotrophic factor content in a rat depression model (Cao et al., 2019; Shen et al., 2020; Deng et al., 2022). Recent investigations also revealed that SNS could produce antidepressant effects by regulating the biosynthesis and metabolism of steroid hormones in the liver (Wang et al., 2020). However, the impact of SNS on adolescent depression and the potential mechanisms governing NAc spine plasticity in such cases had not been examined prior to this study. Our findings indicated that SNS improved depression-like behavior in adolescent mice, reversing the spine abnormalities in the NAc induced by ELS. Additionally, SNS enhanced Rac1 activity, and the inhibition of Rac1 activity weakened the antidepressant effect of SNS. This evidence suggests that SNS executes its antidepressant action by modulating NAc spine remodeling through Rac1. A recent study also demonstrated that the antidepressant effects of SNS were associated with anti-inflammatory benefits (Zong et al., 2019). Moreover, SNS ameliorated depression-like behavior induced by chronic unpredictable mild stress by regulating dendritic spines in the hippocampus via NCOA4-mediated ferritinophagy (Zhang et al., 2023). It is important to note that most studies concerning the antidepressant effect of SNS have been primarily focused on the hippocampus (Zong et al., 2019; Deng et al., 2022; Zhang et al., 2023), and no articles have evaluated the role of the NAc in SNS’s antidepressant effect. To the best of our knowledge, our study is the first to report that Rac1-mediated spine remodeling of NAc plays a crucial role in the antidepressant effect of SNS. However, our study does come with certain limitations. For instance, while numerous studies have underscored the antidepressant-like effects of primary SNS components, including hesperidin, isoglycyrrhizin, glycyrrhizin, paeoniflorin, and saikosaponin A, our investigation did not extensively explore these specific therapeutic constituents in the context of ELS-induced depression. Furthermore, while we have established a connection between Rac1 activity and the observed behavioral and spine anomalies induced by ELS, the extensive role of Rac1 and its interactions with other variables remain to be elucidated. Further investigations are required to explore the depth of the relationship between Rac1 activity, spine abnormalities, and behavioral changes induced by ELS. Additionally, a comprehensive exploration of the distinct therapeutic constituents of SNS and their individual and collective influences on depression-like behaviors is warranted.

In summary, our results provide strong evidence that Rac1 is a critical factor in the pathophysiology of ELS-induced depressive-like behavior in adolescence and SNS exerts its antidepressant action by modulating Rac1 activity and associated spine plasticity in the NAc. This research not only advances understanding of the neurobiological mechanisms underpinning depression induced by early life stress, but also provides evidence for the integration of traditional Chinese medicine into therapeutic approaches.

## Data Availability

The original contributions presented in the study are included in the article/Supplementary Material, further inquiries can be directed to the corresponding authors.
