# Supplementary material for: Si-Ni-San alleviates early life stress-induced depression-like behaviors in adolescence via modulating Rac1 activity and associated spine plasticity in the nucleus accumbens
Source: Front Pharmacol. 2023 Nov 1;14:1274121. doi: 10.3389/fphar.2023.1274121 (PMC10646421; doi:10.3389/fphar.2023.1274121)
Supplement: Supplementary file 1 [file DataSheet1.docx]

**
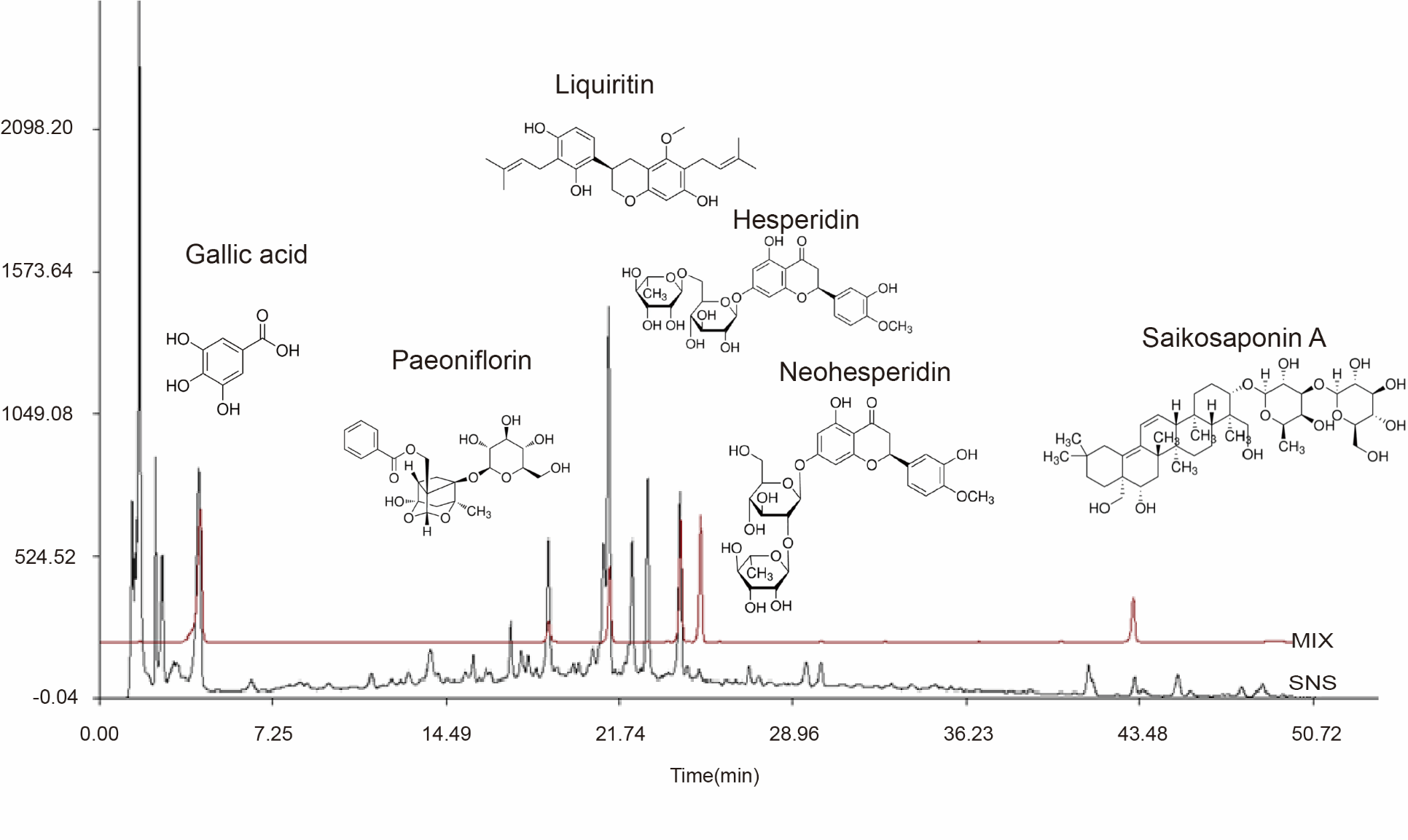
**

**Supplementary Figure 1.** HPLC-based chemoprofile for SNS and some chemical compounds.

HPLC chromatography of SNS：Hesperidin, Liquiritin, Glycyrrhizic acid, Gallic acid, Paeoniflorin and Neohesperidin and chemical structural formulas.

**Supplementary Table 1.** Statistical analysis of Figure1.

|  | | Figure 1B Toatal distance in OFT | | | t(df1, df2)=  value | *P* |
| --- | --- | --- | --- | --- | --- | --- |
|  |  | Control | ELS | |  |  |
| Mean | | 16338.7043 | 16392.3700 | |  |  |
| Std. Deviation | | 4460.7358 | 5598.2479 | |  |  |
| Normality: Shapiro-Wilk normality test | *P* | 0.343 | 0.091 | |  |  |
| Student's t test | |  |  | | t(2,26)=-0.028 | 0.978 |
|  | | Figure 1C Time in center in OFT | | | t(df1, df2)=  value | *P* |
|  |  | Control | | ELS |  |  |
| Mean (cm/s) | | 53.548 | | 46.596 |  |  |
| Std. Deviation | | 33.843 | | 33.819 |  |  |
| Normality: Shapiro-Wilk normality test | *P* | 0.291 | | 0.069 |  |  |
| Student's t test | |  | |  | t(2,26)=0.544 | 0.591 |
|  | | Figure 1D Open arm entries in EPM | | | F(df1, df2)=  value | *P* |
|  |  | Control | | ELS |  |  |
| Mean (cm/s) | | 31.323 | | 30.026 |  |  |
| Std. Deviation | | 11.997 | | 10.231 |  |  |
| Normality: Shapiro-Wilk normality test | *P* | 0.485 | | 0.776 |  |  |
| Student's t test | |  | |  | t(2,26)=0.308 | 0.761 |
|  | | Figure 1E Sucrose consumed in SPT | | |  |  |
|  |  | Control | | ELS |  |  |
| Mean (cm/s) | | 88.085 | | 78.784 |  |  |
| Std. Deviation | | 5.343 | | 7.364 |  |  |
| Normality: Shapiro-Wilk normality test | *P* | 0.865 | | 0.747 |  |  |
| Student's t test | |  | |  | t(2,26)=3.813 | 0.001 |
|  | | Figure 1F Immobility time in TST | | | t(df1, df2)=  value | *P* |
|  |  | Control | ELS | |  |  |
| Mean | | 116.428 | 145.571 | |  |  |
| Std. Deviation | | 32.148 | 18.727 | |  |  |
| Normality: Shapiro-Wilk normality test | *P* | 0.475 | 0.420 | |  |  |
| Student's t test | |  |  | | t(2,26)=-2.931 | 0.007 |
|  | | Figure 1G Immobility time in FST | | | t(df1, df2)=  value | *P* |
|  |  | Control | | ELS |  |  |
| Mean | | 98.785 | | 143.642 |  |  |
| Std. Deviation | | 55.413 | | 25.193 |  |  |
| Normality: Shapiro-Wilk normality test | *P* | 0.271 | | 0.504 |  |  |
| Student's t test | |  | |  | t(2,26)=-2.757 | 0.011 |

**Supplementary Table 2.** Statistical analysis of Figure2.

|  | | Figure 2C Total spine density | | | t(df1, df2)=  value | *P* |
| --- | --- | --- | --- | --- | --- | --- |
|  |  | Control | | ELS |  |  |
| Mean | | 14.287 | | 15.654 |  |  |
| Std. Deviation | | 1.906 | | 2.451 |  |  |
| Normality: Shapiro-Wilk normality test | *P* | 0.986 | | 0.773 |  |  |
| Student's t test | |  | |  | t(2,48)=-2.202 | 0.033 |
|  | | Figure 2D Thin spine density | | | t(df1, df2)=  value | *P* |
|  |  | Control | | ELS |  |  |
| Mean (cm/s) | | 8.926 | | 8.928 |  |  |
| Std. Deviation | | 1.914 | | 0.2.053 |  |  |
| Normality: Shapiro-Wilk normality test | *P* | 0.182 | | 0.084 |  |  |
| Student's t test | |  | |  | t(2,48)=-0.004 | 0.997 |
|  | | Figure 2E Mushroom spine density | | | t(df1, df2)=  value | *P* |
|  |  | Control | ELS | |  |  |
| Mean | | 3.354 | 2.748 | |  |  |
| Std. Deviation | | 1.170 | 0.812 | |  |  |
| Normality: Shapiro-Wilk normality test | *P* | 0.174 | 0.545 | |  |  |
| Student's t test | |  |  | | t(2,48)=2.219 | 0.039 |
|  | | Figure 2F Stubby spine density | | | t(df1, df2)=  value | *P* |
|  |  | Control | | ELS |  |  |
| Mean (cm/s) | | 2.252 | | 4.037 |  |  |
| Std. Deviation | | 0.918 | | 0.879 |  |  |
| Normality: Shapiro-Wilk normality test | *P* | 0.441 | | 0.255 |  |  |
| Student's t test | |  | |  | t(2,48)=-6.629 | <0.001 |

**Supplementary Table 3.** Statistical analysis of Figure3.

|  | | Figure 3B relative Rac1-GTP/Rac1-Total protein level | | | | | | | | | | t(df1, df2)=  value | | | | *P* |
| --- | --- | --- | --- | --- | --- | --- | --- | --- | --- | --- | --- | --- | --- | --- | --- | --- |
|  |  | Control | | | | | ELS | | | | |  |  |  |  |  |
| Mean | | 1.516 | | | | | 0.9640 | | | | |  | | | |  |
| Std. Deviation | | 0.37 | | | | | 0.30 | | | | |  | | | |  |
| Normality: Shapiro-Wilk normality test | *P* | 0.9452 | | | | | 0.6721 | | | | |  | | | |  |
| Student's t test | |  | | | | |  | | | | | t(2,8)=2.582 | | | | 0.033 |
|  | | Figure 3C relative p-Pak/Pak protein level | | | | | | | | | | t(df1, df2)=  value | | | | *P* |
|  |  | Control | | | | | | ELS | | | |  |  |  |  |  |
| Mean (cm/s) | | 1.29 | | | | | | 0.95 | | | |  | | | |  |
| Std. Deviation | | 0.20 | | | | | | 0.09 | | | |  | | | |  |
| Normality: Shapiro-Wilk normality test | *P* | 0.847 | | | | | | 0.947 | | | |  | | | |  |
| Student's t test | |  | | | | | |  | | | | t(2,8)=3.376 | | | | 0.01 |
|  | | Figure 3F relative Rac1-GTP/Rac1-Total protein level | | | | | | | | | | F(df1, df2)=  value | | *P* | | *Bonferroni* |
|  |  | eGFP | | Rac1-CA | | | | | Rac1-DN | | |  | |  | |  |
| Mean | | 1.09 | | 1.38 | | | | | 0.73 | | |  | |  | |  |
| Std. Deviation | | 0.12 | | 0.14 | | | | | 0.19 | | |  | |  | |  |
| Normality: Shapiro-Wilk normality test | *P* | 0.993 | | 0.399 | | | | | 0.905 | | |  | |  | |  |
| Homogeneity of variances test: Levene Statistic | |  | |  | | | | |  | | | F(3,9)=0.338 | | 0.722 | |  |
| One way ANOVA | |  | |  | | | | |  | | | F(3,9)=46.277 | | <0.001 | |  |
| eGFP vs. Rac1-CA | |  | |  | | | | |  | | |  | |  | | 0.043 |
| eGFP vs. Rac1-DN | |  | |  | | | | |  | | |  | |  | | 0.015 |
|  | | Figure 3G relative p-Pak/Pak protein level | | | | | | | | | | F(df1, df2)=  value | | *P* | | *Bonferroni* |
|  |  | eGFP | | Rac1-CA | | | | | Rac1-DN | | |  | |  | |  |
| Mean | | 1.05 | | 1.31 | | | | | 0.74 | | |  | |  | |  |
| Std. Deviation | | 0.10 | | 0.07 | | | | | 0.09 | | |  | |  | |  |
| Normality: Shapiro-Wilk normality test | *P* | 0.647 | | 0.629 | | | | | 0.210 | | |  | |  | |  |
| Homogeneity of variances test: Levene Statistic | |  | |  | | | | |  | | | F(3,9)=0.250 | | 0.784 | |  |
| One way ANOVA | |  | |  | | | | |  | | | F(3,9)=18.429 | | <0.001 | |  |
| eGFP vs. Rac1-CA | |  | |  | | | | |  | | |  | |  | | 0.044 |
| eGFP vs. Rac1-DN | |  | |  | | | | |  | | |  | |  | | 0.015 |
|  | | Figure 3H Sucrose consumed in SPT | | | | | | | | | | | F=  value | | *P* | *Bonferroni* |
|  |  | eGFP-Control | Rac1(CA)-Control | | Rac1(DN)-Control | eGFP-ELS | | | | Rac1(CA)-ELS | Rac1(DN)-ELS | |  | |  |  |
| Mean | | 83.30 | 82.77 | | 75.39 | 72.50 | | | | 83.78 | 73.51 | |  | |  |  |
| Std. Deviation | | 6.75 | 2.88 | | 5.24 | 7.23 | | | | 4.71 | 7.07 | |  | |  |  |
| Normality: Shapiro-Wilk normality test | *P* | 0.154 | 0.578 | | 0.332 | 0.123 | | | | 0.822 | 0.473 | |  | |  |  |
| Homogeneity of variances test: Levene Statistic | |  | | | | | | | | | | | F(5,42)=0.338 | | 0.340 |  |
| Two way ANOVA | | Virus treatment | | | | | | | | | | | 9.216 | | <0.001 |  |
|  |  | ELS treatment | | | | | | | | | | | 5.298 | | 0.026 |  |
|  |  | Virus*ELS treatment | | | | | | | | | | | 4.424 | | 0.018 |  |
| eGFP-Control vs. Rac1(DN)-Control | |  | | | | | | | | | | |  | |  | 0.030 |
| eGFP-Control vs. eGFP-ELS | |  | | | | | | | | | | |  | |  | 0.001 |
| eGFP-ELS vs. Rac1(CA)-ELS | |  | | | | | | | | | | |  | |  | 0.001 |
|  | | Figure 3I Immobility in TST | | | | | | | | | | | F=  value | | *P* | *Bonferroni* |
|  |  | eGFP-Control | Rac1(CA)-Control | | Rac1(DN)-Control | eGFP-ELS | | | | Rac1(CA)-ELS | Rac1(DN)-ELS | |  | |  |  |
| Mean | | 100.63 | 121.50 | | 158.38 | 138.88 | | | | 113.63 | 155.00 | |  | |  |  |
| Std. Deviation | | 19.99 | 24.83 | | 18.50 | 13.93 | | | | 21.94 | 17.86 | |  | |  |  |
| Normality: Shapiro-Wilk normality test | *P* | 0.809 | 0.137 | | 0.764 | 0.502 | | | | 0.523 | 0.589 | |  | |  |  |
| Homogeneity of variances test: Levene Statistic | |  | | | | | | | | | | | F(5,42)=1.170 | | 0.711 |  |
| Two way ANOVA | | Virus treatment | | | | | | | | | | | 19.257 | | <0.001 |  |
|  |  | ELS treatment | | | | | | | | | | | 2.130 | | 0.123 |  |
|  |  | Virus*ELS treatment | | | | | | | | | | | 7.238 | | 0.003 |  |
| eGFP-Control vs. Rac1(DN)-Control | |  | | | | | | | | | | |  | |  | <0.001 |
| eGFP-Control vs. eGFP-ELS | |  | | | | | | | | | | |  | |  | <0.001 |
| eGFP-ELS vs. Rac1(CA)-ELS | |  | | | | | | | | | | |  | |  | 0.038 |
|  | | Figure 3J Immobility in FST | | | | | | | | | | | F=  value | | *P* | *Bonferroni* |
|  |  | eGFP-Control | Rac1(CA)-Control | | Rac1(DN)-Control | eGFP-ELS | | | | Rac1(CA)-ELS | Rac1(DN)-ELS | |  | |  |  |
| Mean | | 83.30 | 82.77 | | 75.39 | 72.50 | | | | 83.78 | 73.51 | |  | |  |  |
| Std. Deviation | | 6.75 | 2.88 | | 5.24 | 7.23 | | | | 4.71 | 7.07 | |  | |  |  |
| Normality: Shapiro-Wilk normality test | *P* | 0.117 | 0.427 | | 0.323 | 0.803 | | | | 0.993 | 0.410 | |  | |  |  |
| Homogeneity of variances test: Levene Statistic | |  | | | | | | | | | | | F(5,42)=1.224 | | 0.315 |  |
| Two way ANOVA | | Virus treatment | | | | | | | | | | | 7.786 | | 0.001 |  |
|  |  | ELS treatment | | | | | | | | | | | 7.684 | | 0.008 |  |
|  |  | Virus*ELS treatment | | | | | | | | | | | 6.220 | | 0.004 |  |
| eGFP-Control vs. Rac1(DN)-Control | |  | | | | | | | | | | |  | |  | 0.002 |
| eGFP-Control vs. eGFP-ELS | |  | | | | | | | | | | |  | |  | <0.001 |
| eGFP-ELS vs. Rac1(CA)-ELS | |  | | | | | | | | | | |  | |  | 0.006 |

**Supplementary Table 4.** Statistical analysis of Figure4.

|  | | Figure 4B Total spine density | | | | | | F=  value | *P* | *Bonferroni* |
| --- | --- | --- | --- | --- | --- | --- | --- | --- | --- | --- |
|  |  | eGFP-Control | Rac1(CA)-Control | Rac1(DN)-Control | eGFP-ELS | Rac1(CA)-ELS | Rac1(DN)-ELS |  |  |  |
| Mean | | 14.82 | 15.00 | 16.49 | 16.68 | 15.15 | 16.63 |  |  |  |
| Std. Deviation | | 2.313 | 2.028 | 1.450 | 2.051 | 1.904 | 1.861 |  |  |  |
| Normality: Shapiro-Wilk normality test | *P* | 0.4998 | 0.6072 | 0.3916 | 0.2056 | 0.8592 | 0.1721 |  |  |  |
|  | |  | | | | | | F(5,142) |  |  |
| Two way ANOVA | | Virus treatment | | | | | | 7.162 | 0.001 |  |
|  |  | ELS treatment | | | | | | 4.989 | 0.027 |  |
|  |  | Virus*ELS treatment | | | | | | 3.202 | 0.044 |  |
| eGFP-Control vs. Rac1(DN)-Control | |  | | | | | |  |  | 0.009 |
| eGFP-Control vs. eGFP-ELS | |  | | | | | |  |  | 0.010 |
| eGFP-ELS vs. Rac1(CA)-ELS | |  | | | | | |  |  | 0.021 |
|  | | Figure 4C Thin spine density | | | | | | F=  value | *P* | *Bonferroni* |
|  |  | eGFP-Control | Rac1(CA)-Control | Rac1(DN)-Control | eGFP-ELS | Rac1(CA)-ELS | Rac1(DN)-ELS |  |  |  |
| Mean | | 8.626 | 9.626 | 10.00 | 9.987 | 8.901 | 10.43 |  |  |  |
| Std. Deviation | | 1.945 | 2.873 | 1.676 | 2.518 | 1.976 | 1.928 |  |  |  |
| Normality: Shapiro-Wilk normality test | *P* | 0.8366 | 0.8249 | 0.7698 | 0.0961 | 0.5906 | 0.0563 |  |  |  |
|  | |  | | | | | | F(5,142) |  |  |
| Two way ANOVA | | Virus treatment | | | | | | 2.963 | 0.055 |  |
|  |  | ELS treatment | | | | | | .972 | 0.326 |  |
|  |  | Virus*ELS treatment | | | | | | 2.802 | 0.064 |  |
| eGFP-Control vs. Rac1(DN)-Control | |  | | | | | |  |  | 0.085 |
| eGFP-Control vs. eGFP-ELS | |  | | | | | |  |  | 0.076 |
| eGFP-ELS vs. Rac1(CA)-ELS | |  | | | | | |  |  | 0.256 |
|  | | Figure 4D Mushroom spine density | | | | | | F=  value | *P* | *Bonferroni* |
|  |  | eGFP-Control | Rac1(CA)-Control | Rac1(DN)-Control | eGFP-ELS | Rac1(CA)-ELS | Rac1(DN)-ELS |  |  |  |
| Mean | | 4.022 | 3.978 | 2.914 | 2.991 | 4.114 | 2.677 |  |  |  |
| Std. Deviation | | 0.9548 | 1.499 | 0.8329 | 0.9872 | 1.245 | 0.6782 |  |  |  |
| Normality: Shapiro-Wilk normality test | *P* | 0.7633 | 0.1892 | 0.3914 | 0.9922 | 0.1701 | 0.7256 |  |  |  |
|  | |  | | | | | | F(5,142) |  |  |
| Two way ANOVA | | Virus treatment | | | | | | 16.477 | <0.01 |  |
|  |  | ELS treatment | | | | | | 4.845 | 0.029 |  |
|  |  | Virus*ELS treatment | | | | | | 3.796 | 0.025 |  |
| eGFP-Control vs. Rac1(DN)-Control | |  | | | | | |  |  | 0.001 |
| eGFP-Control vs. eGFP-ELS | |  | | | | | |  |  | <0.001 |
| eGFP-ELS vs. Rac1(CA)-ELS | |  | | | | | |  |  | <0.001 |
|  | | Figure 4E Stubby spine density | | | | | | F=  value | *P* | *Bonferroni* |
|  |  | eGFP-Control | Rac1(CA)-Control | Rac1(DN)-Control | eGFP-ELS | Rac1(CA)-ELS | Rac1(DN)-ELS |  |  |  |
| Mean | | 2.170 | 2.294 | 3.385 | 3.699 | 2.056 | 3.523 |  |  |  |
| Std. Deviation | | 0.6580 | 0.8739 | 0.8088 | 1.454 | 0.7429 | 0.8620 |  |  |  |
| Normality: Shapiro-Wilk normality test | *P* | 0.2577 | 0.4741 | 0.1431 | 0.5826 | 0.5723 | 0.4606 |  |  |  |
|  | |  | | | | | | F(5,142) |  |  |
| Two way ANOVA | | Virus treatment | | | | | | 23.034 | <0.001 |  |
|  |  | ELS treatment | | | | | | 9.565 | 0.002 |  |
|  |  | Virus*ELS treatment | | | | | | 12.232 | <0.001 |  |
| eGFP-Control vs. Rac1(DN)-Control | |  | | | | | |  |  | <0.001 |
| eGFP-Control vs. eGFP-ELS | |  | | | | | |  |  | <0.001 |
| eGFP-ELS vs. Rac1(CA)-ELS | |  | | | | | |  |  | <0.001 |

**Supplementary Table 5.** Statistical analysis of Figure5.

|  | | Figure 5B Sucrose consumed in SPT | | | | | | | | | F(df1, df2)=  value | *P* | *Bonferroni* |
| --- | --- | --- | --- | --- | --- | --- | --- | --- | --- | --- | --- | --- | --- |
|  |  | Control | ELS | | Positive | SNS-L | | SNS-M | | SNS-H |  |  |  |
| Mean | | 84.61 | 70.76 | | 84.59 | 82.06 | | 83.16 | | 83.34 |  |  |  |
| Std. Deviation | | 8.46 | 11.54 | | 7.10 | 8.65 | | 6.75 | | 5.48 |  |  |  |
| Normality: Shapiro-Wilk normality test | *P* | 0.200 | 0.845 | | 0.699 | 0.212 | | 0.284 | | 0.312 |  |  |  |
| Homogeneity of variances test: Levene Statistic | |  | | | | | | | | | F(5,78)=0.428 | 0.828 |  |
| One way ANOVA | |  | | | | | | | | | F(5,78)=5.837 | <0.001 |  |
| Control vs. ELS | |  | | | | | | | | |  |  | <0.001 |
| Positive vs. ELS | |  | | | | | | | | |  |  | <0.001 |
| SNS-L vs. ELS | |  | | | | | | | | |  |  | 0.002 |
| SNS-M vs. ELS | |  | | | | | | | | |  |  | 0.001 |
| SNS-H vs. ELS | |  | | | | | | | | |  |  | 0.001 |
|  | | Figure 5C Immobility time in TST | | | | | | | | | F(df1, df2)=  value | *P* | *Bonferroni* |
|  |  | Control | ELS | | Positive | SNS-L | | SNS-M | | SNS-H |  |  |  |
| Mean | | 101.00 | 143.29 | | 104.86 | 97.86 | | 109.07 | | 106.00 |  |  |  |
| Std. Deviation | | 19.61 | 30.83 | | 25.56 | 27.08 | | 35.00 | | 26.52 |  |  |  |
| Normality: Shapiro-Wilk normality test | 0.509 | 0.5088 | 0.0502 | | 0.5334 | 0.7378 | | 0.1265 | | 0.8758 |  |  |  |
| Homogeneity of variances test: Levene Statistic | |  | | | | | | | | | F(5,78)=0.669 | 0.648 |  |
| One way ANOVA | | ­­- | | | | | | | | | F(5,78)=4.983 | <0.001 |  |
| Control vs. ELS | |  | | | | | | | | |  |  | 0.001 |
| Positive vs. ELS | |  | | | | | | | | |  |  | 0.002 |
| SNS-L vs. ELS | |  | | | | | | | | |  |  | <0.001 |
| SNS-M vs. ELS | |  | | | | | | | | |  |  | 0.008 |
| SNS-H vs. ELS | |  | | | | | | | | |  |  | 0.001 |
|  | | Figure 5D Immobility time in FST | | | | | | | | | F(df1, df2)=  value | *P* | *Bonferroni* |
|  |  | Control | ELS | | Positive | SNS-L | | SNS-M | | SNS-H |  |  |  |
| Mean | | 100.21 | 149.57 | | 113.71 | 129.64 | | 114.57 | | 108.57 |  |  |  |
| Std. Deviation | | 26.85 | 25.11 | | 29.04 | 28.78 | | 20.83 | | 45.37 |  |  |  |
| Normality: Shapiro-Wilk normality test | *P* | 0.062 | 0.321 | | 0.131 | 0.974 | | 0.359 | | 0.077 |  |  |  |
| Homogeneity of variances test: Levene Statistic | |  | | | | | | | | | F(5,78)=0.868 | 0.507 |  |
| One way ANOVA | |  | | | | | | | | | F(5,78)=4,742 | 0.507 |  |
| Control vs. ELS | |  | | | | | | | | |  |  | <0.001 |
| Positive vs. ELS | |  | | | | | | | | |  |  | 0.012 |
| SNS-L vs. ELS | |  | | | | | | | | |  |  | 0.325 |
| SNS-M vs. ELS | |  | | | | | | | | |  |  | 0.015 |
| SNS-H vs. ELS | |  | | | | | | | | |  |  | 0.003 |
|  | | Figure 5F Total spine density | | | | | | | | | K | *P* | *Bonferroni* |
|  |  | Control | | ELS | | | Positive | | SNS | |  |  |  |
| Mean | | 13.68 | | 15.61 | | | 13.31 | | 13.76 | |  |  |  |
| Std. Deviation | | 2.761 | | 2.531 | | | 2.915 | | 2.785 | |  |  |  |
| Normality: Shapiro-Wilk normality test | *P* | 0.7276 | | 0.1464 | | | 0.3384 | | 0.6733 | |  |  |  |
| Homogeneity of variances test : Levene Statistic | |  | | | | | | | | |  |  |  |
| One way ANOVA | |  | | | | | | | | | F (3, 109) = 4.019 | 0.001 |  |
| Control vs. ELS | |  | | | | | | | | |  |  | 0.0283 |
| Positive vs. ELS | |  | | | | | | | | |  |  | 0.0068 |
| SNS vs. ELS | |  | | | | | | | | |  |  | 0.0352 |
|  | | Figure 5G Thin spine density | | | | | | | | | F(df1, df2)=  value | *P* | *Bonferroni* |
|  |  | Control | | ELS | | | Positive | | SNS | |  |  |  |
| Mean | | 8.243 | | 9.278 | | | 7.923 | | 7.849 | |  |  |  |
| Std. Deviation | | 2.533 | | 2.333 | | | 2.645 | | 2.424 | |  |  |  |
| Normality: Shapiro-Wilk normality test | *P* | 0.4046 | | 0.3427 | | | 0.1761 | | 0.1230 | |  |  |  |
| Homogeneity of variances test : Levene Statistic | |  | | | | | | | | | F(3,113)=0.927 | 0.430 |  |
| One way ANOVA | | - | | | | | | | | | F (3, 109) = 2.026 | 0.1146 |  |
| Control vs. ELS | |  | | | | | | | | |  |  | 0.3556 |
| Positive vs. ELS | |  | | | | | | | | |  |  | 0.1311 |
| SNS vs. ELS | |  | | | | | | | | |  |  | 0.0915 |
|  | | Figure 5H Mushroom spine density | | | | | | | | | F(df1, df2)=  value | *P* | *Bonferroni* |
|  |  | Control | | ELS | | | Positive | | SNS | |  |  |  |
| Mean | | 3.541 | | 2.687 | | | 3.342 | | 3.449 | |  |  |  |
| Std. Deviation | | 1.365 | | 0.8574 | | | 0.6946 | | 0.9838 | |  |  |  |
| Normality: Shapiro-Wilk normality test | *P* | 0.0874 | | 0.8321 | | | 0.8243 | | 0.9024 | |  |  |  |
| Homogeneity of variances test : Levene Statistic | |  | | | | | | | | |  |  |  |
| One way ANOVA | |  | | | | | | | | | F (3, 108) = 4.259 | 0.007 |  |
| Control vs. ELS | |  | | | | | | | | |  |  | 0.0057 |
| Positive vs. ELS | |  | | | | | | | | |  |  | 0.0487 |
| SNS vs. ELS | |  | | | | | | | | |  |  | 0.0138 |
|  | | Figure 5I Stubby spine density | | | | | | | | | F(df1, df2)=  value | *P* | *Bonferroni* |
|  |  | Control | | ELS | | | Positive | | SNS | |  |  |  |
| Mean | | 2.699 | | 3.374 | | | 2.098 | | 2.461 | |  |  |  |
| Std. Deviation | | 0.7831 | | 0.9854 | | | 0.7485 | | 0.6142 | |  |  |  |
| Normality: Shapiro-Wilk normality test | *P* | 0.1501 | | 0.8404 | | | 0.0612 | | 0.6735 | |  |  |  |
| Homogeneity of variances test : Levene Statistic | |  | | | | | | | | | F(3,121)=0.916 | 0.435 |  |
| One way ANOVA | |  | | | | | | | | | F (3, 108) = 12.74 | <0.001 |  |
| Control vs. ELS | |  | | | | | | | | |  |  | 0.005 |
| Positive vs. ELS | |  | | | | | | | | |  |  | <0.001 |
| SNS vs. ELS | |  | | | | | | | | |  |  | <0.001 |

**Supplementary Table 6.** Statistical analysis of Figure6.

|  | | Figure 6B relative Rac1-GTP/Rac1-Total protein level | | | | F(df1, df2)=  value | *P* | *Bonferroni* |
| --- | --- | --- | --- | --- | --- | --- | --- | --- |
|  |  | Control | ELS | Positive | SNS |  |  |  |
| Mean | | 1.48 | 0.93 | 1.38 | 1.38 |  |  |  |
| Std. Deviation | | 0.21 | 0.33 | 0.15 | 0.27 |  |  |  |
| Normality: Shapiro-Wilk normality test | *P* | 0.324 | 0.108 | 0.273 | 0.849 |  |  |  |
| Homogeneity of variances test : Levene Statistic | |  | | | | F(3,12)= 1.282 | 0.309 |  |
| One way ANOVA | |  | | | | F(3,12)=5.548 | 0.006 |  |
| Control vs. ELS | |  | | | |  |  | 0.004 |
| Positive vs. ELS | |  | | | |  |  | 0.023 |
| SNS vs. ELS | |  | | | |  |  | 0.016 |
|  | | Figure 6C relative p-Pak/Pak protein level | | | | F(df1, df2)=  value | *P* | *Bonferroni* |
|  |  | Control | ELS | Positive | SNS |  |  |  |
| Mean | | 1.38 | 0.93 | 1.37 | 1.41 |  |  |  |
| Std. Deviation | | 0.32 | 0.12 | 0.28 | 0.32 |  |  |  |
| Normality: Shapiro-Wilk normality test | *P* | 0.379 | 0.168 | 0.277 | 0.568 |  |  |  |
| Homogeneity of variances test : Levene Statistic | |  | | | | F(3,12)=0.767 | 0.526 |  |
| One way ANOVA | | - | | | | F(3,12)=4.398 | 0.016 |  |
| Control vs. ELS | |  | | | |  |  | 0.022 |
| Positive vs. ELS | |  | | | |  |  | 0.035 |
| SNS vs. ELS | |  | | | |  |  | 0.015 |
|  | | Figure 6D Sucrose consumed in SPT | | | | F(df1, df2)=  value | *P* | *Bonferroni* |
|  |  | Control | ELS | SNS | SNS-Rac1(DN) |  |  |  |
| Mean | | 85.3506 | 72.6589 | 84.7639 | 76.2803 |  |  |  |
| Std. Deviation | | 7.41552 | 8.05585 | 6.49028 | 7.27524 |  |  |  |
| Normality: Shapiro-Wilk normality test | *P* | 0.123 | 0.251 | 0.395 | 0.677 |  |  |  |
| Homogeneity of variances test : Levene Statistic | |  | | | | F(3,28)=0.226 | 0.877 |  |
| One way ANOVA | |  | | | | F(3,28)=5.897 | 0.003 |  |
| Control vs. ELS | |  | | | |  |  | 0.005 |
| SNS vs. ELS | |  | | | |  |  | 0.007 |
| SNS vs. SNS-Rac1(DN) | |  | | | |  |  | 0.016 |
|  | | Figure 6E Immobility time in TST | | | | F(df1, df2)=  value | *P* | *Bonferroni* |
|  |  | Control | ELS | SNS | SNS-Rac1(DN) |  |  |  |
| Mean | | 111.5825 | 154.8737 | 115.3750 | 146.1314 |  |  |  |
| Std. Deviation | | 22.78075 | 19.62479 | 30.09510 | 21.07866 |  |  |  |
| Normality: Shapiro-Wilk normality test | *P* | 0.3698 | 0.6961 | 0.9908 | 0.4820 |  |  |  |
| Homogeneity of variances test : Levene Statistic | |  | | | | F(3,28)=2.219 | 0.108 |  |
| One way ANOVA | |  | | | | F(3,28)=9.549 | 0.002 |  |
| Control vs. ELS | |  | | | |  |  | 0.004 |
| SNS vs. ELS | |  | | | |  |  | <0.001 |
| SNS vs. SNS-Rac1(DN) | |  | | | |  |  | 0.018 |
|  | | Figure 6F Immobility time in FST | | | | F(df1, df2)=  value | *P* | *Bonferroni* |
|  |  | Control | ELS | SNS | SNS-Rac1(DN) |  |  |  |
| Mean | | 111.582 | 154.873 | 115.375 | 145.865 |  |  |  |
| Std. Deviation | | 22.780 | 19.624 | 30.095 | 19.529 |  |  |  |
| Normality: Shapiro-Wilk normality test | *P* | 0.2352 | 0.4391 | 0.7781 | 0.3687 |  |  |  |
| Homogeneity of variances test : Levene Statistic | |  | | | | F(3,28)=0.950 | 0.430 |  |
| One way ANOVA | | - | | | | F(3,28)=6.857 | 0.001 |  |
| Control vs. ELS | |  | | | |  |  | 0.005 |
| SNS vs. ELS | |  | | | |  |  | 0.011 |
| SNS vs. SNS-Rac1(DN) | |  | | | |  |  | 0.038 |
